# Hospitalization Trends in Adult Patients with COPD and Other Respiratory Diseases in Northeast China from 2005 to 2015

**DOI:** 10.1155/2018/1060497

**Published:** 2018-02-08

**Authors:** Honglei Liu, Ni Wang, Wei Chen, Wenyan Liu, Shiping Wang, Jianbo Lei, Hui Chen

**Affiliations:** ^1^School of Biomedical Engineering, Capital Medical University, Beijing, China; ^2^Beijing Key Laboratory of Fundamental Research on Biomechanics in Clinical Application, Capital Medical University, Beijing, China; ^3^Department of Respiratory, Ruijin Hospital, School of Medicine, Shanghai Jiaotong University, Shanghai, China; ^4^Health Information Center, Dalian, Liaoning, China; ^5^Health Science Center, Peking University, Beijing, China

## Abstract

Chronic obstructive pulmonary disease (COPD), pneumonia, asthma, and lung cancer are four common respiratory diseases that impose a substantial economic burden on both patients and government in China. The objective of our study is to analyze the temporal trends of several clinical tracking metrics for hospitalization regarding these diseases. Hospital discharge data of 54 hospitals for the period 2005–2015 were derived from the Health and Family Planning Commission in Northeast China. The age-adjusted rate of discharge for the four respiratory diseases increased significantly (COPD, pneumonia, asthma: *P* trend <  .001; lung cancer: *P* trend =  .046). The mean LOS for the four diseases all showed a significant decline (*P* trend <  .001), whereas the mean charge per stay and aggregate charge followed an upward trend over time (*P* trend <  .001). There was a clear upward trend for the readmission rate for asthma patients (*P* trend =  .001), while the trend for COPD patients was unclear (*P* trend =  .224). Age-adjusted discharge rates, LOS, and charges for hospitalization regarding several common respiratory diseases in China showed different patterns of change over the past decade. Our results should aid government and administrators in making informed decisions about the management and treatment of respiratory diseases.

## 1. Introduction

Despite efforts to encourage smoking cessation and improve air quality, respiratory diseases still represent a serious public health problem worldwide, with high morbidity and mortality and a heavy economic toll, especially chronic obstructive pulmonary disease (COPD) [1–5]. Although the chief cause of COPD is cigarette smoking [6, 7] which led to approximately 80% of deaths from COPD from 2000 to 2004, [8] other etiology includes indoor air pollution, recurring respiratory infections, and asthma. [9, 10]. Patients who suffer from COPD may experience cough, dyspnea, chest tightness, and wheezing [11, 12]. As lung function declines, patients may encounter difficulties in daily living. COPD promises to remain a major public health problem in the 21st century [13]. Several statistical analyses of clinical metrics of COPD to discern the temporal trend have been published [10, 14–17].

Lung cancer, pneumonia, and asthma are three other prevailing respiratory diseases, with asthma being almost as common as COPD [1, 18]. Pneumonia is the most common lower respiratory tract infection, usually caused by bacteria. The many factors that increase the risk of lung cancer include radon gas, outdoor air pollution, and smoking [19–21], although smoking is the major contributory factor.

All of these diseases have constituted a substantially increasing economic burden for both patients and governments, especially in the developing countries. To obtain a clearer picture of the economic burden with respect to hospitalization, we analyzed the temporal trends of several clinical tracking metrics, such as hospital discharge rate, length of stay (LOS), charge per stay, and readmission rate, which may be used to estimate the economic burden, learn the current medical treatment level, and explore the disease development tendency. We collected the electronic medical records of all hospitals in Dalian city and then analyzed the metrics for patients aged 18 years and older diagnosed with COPD, lung cancer, pneumonia, or asthma during the period 2005–2015.

## 2. Materials and Methods

We collected more than 6 million medical records of hospitals in Dalian, the second largest city in Northeast China, with almost 7 million permanent residents, from 2005 to 2015. Patients aged ≥18 years with COPD, pneumonia, asthma, or lung cancer as principal diagnosis were chosen for analysis. The basic information of each patient in the electronic medical records included demographic information (sex and date of birth), admission date, discharge date, principal diagnosis, and up to 5 secondary diagnoses. The data was authorized by the Information Center, Health and Family Planning Commission of Dalian Municipality. Patients' personal information, such as name, ID number, address, phone number, and zip code, was all removed from the dataset before we could access the data remotely. Therefore, the data was used in an anonymous and safe manner.

We used International Classification of Diseases, 10th Revision (ICD-10) [22] codes, to identify diseases and then used Clinical Classifications Software (CCS) for ICD-10 [23–25] to recode the codes into categorization codes 1–259. CCS provided a method for presenting more clinically meaningful categories for diagnoses which had been successfully applied in statistical reports with specific conditions. The CSS code of COPD is 127, which includes the following ICD-10 codes: J40, J41.0, J41.1, J41.8, J42, J43.0, J43.1, J43.2, J43.8, J43.9, J44.0, J44.1, J44.9, J47.0, J47.1, and J47.9. The CCS codes of lung cancer, pneumonia, and asthma are 19 (ICD-10 codes: C34.0, C34.1, C34.2, C34.3, C34.8, C34.9, D02.2, and Z85.1), 122 (ICD-10 codes: A01.0, A02.2, A20.2, A21.2, A22.1, A31.0, A37.0, A37.1, A43.0, A48.1, B01.2, B05.2, B37.1, B38.0, B38.1, B38.2, B39.0, B39.1, B39.2, B58.3, B59, B77.8, J12.0, J12.1, J12.2, J12.3, J12.8, J12.9, J13, J14, J15.0, J15.1, J15.2, J15.3, J15.4, J15.5, J15.6, J15.7, J15.8, J15.9, J16.0, J16.8, J17, J18.0, J18.1, J18.8, J18.9, J85.1, and J85.2), and 128 (ICD-10 codes: J45.2, J45.3, J45.4, J45.4, J45.5, and J45.9), respectively.

To explore the relationship between clinical metrics and age, we categorized patients into three age groups: 18–35 years, 36–60 years, and >60 years. We divided the number of hospitalizations by population number of the corresponding age group (population data were obtained from the Dalian Statistical Yearbook, available online at http://www.stats.dl.gov.cn/) to compute the discharge rate. For clinical tracking metrics, such as hospital discharge rate, LOS, charge per stay, and readmission rate, we performed linear regression to estimate the mean rate of change per year. Readmission rate was identified when the CCS codes of readmission principal diagnosis remained the same within 30 days. Costs were the total charge for the patient's entire stay in hospital. All metrics were age-adjusted to the projected year 2005 Dalian population by using three age groups (18–35 years, 36–60 years, and >60 years).

We also calculated Charlson Comorbidity Index (CCI) [26] for each patient to explore the effect of comorbidities on hospitalization number, mean LOS, mean charge per stay, and readmission rate. CCI could predict in-hospital or short-term mortality [27–30], which took the presence of 17 different kinds of conditions into account, such as pulmonary disease, renal disease, liver disease, diabetes mellitus, and peripheral arterial disease. Weights 1, 2, 3, or 6 were assigned to different Charlson comorbidities, and CCI score was then obtained by a summation of all Charlson comorbidity weights. We then divided patients into three groups according to CCI score, without comorbidity (CCI score = 0), mild comorbidities (CCI score = 1), and serious comorbidities (CCI score ≥ 2), to represent the different degree of effect on in-hospital metrics.

## 3. Results

**Figure 1:**
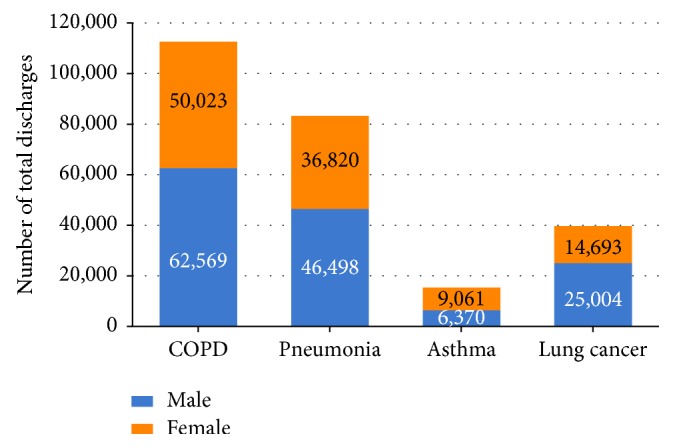
Number of hospitalizations for patients aged ≥18 years with COPD, pneumonia, asthma, and lung cancer, in Dalian city, from 2005 to 2013.

The number of total hospitalizations in Dalian increased significantly, from 351,754 in 2005 to 955,713 in 2015 (*P* trend <  .001), and those for adult patients ranged from 321,478 (91.4%) in 2005 to 885,771 (92.7%) in 2015. Among the four studied respiratory diseases, the number of hospitalizations for adult patients diagnosed with COPD always occupied first place every year from 2005 to 2015, whereby male patients accounted for 55.6% of 112,592 hospitalizations. Number of hospitalizations for patients diagnosed with pneumonia, asthma, and lung cancer were 83,318, 15,431, and 39,697, respectively, whereby men accounted for 55.8%, 41.3%, and 63.0%, respectively (Table S1 and Figure 1).

**Figure 2:**
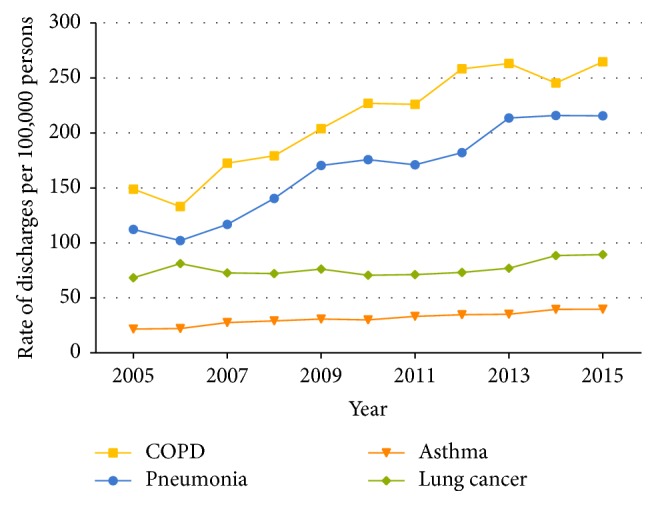
Age-adjusted discharge rate for hospitalized patients aged ≥18 years with COPD, pneumonia, asthma, and lung cancer, in Dalian city, from 2005 to 2015.

For COPD, the age-adjusted discharge rate ranged from 149.0 per 100,000 population in 2005 to 264.7 per 100,000 population in 2015, a significant improvement (13.5 per 100,000 population per year, 95% confidence interval [CI]: 10.2–16.7 per 100,000 population per year, *P* trend <  .001). Among the 3 age groups, all discharge rates showed a clear upward trend (*P* trend <  .001), especially for patients aged >60 years, showing the most rapid improvement from 647.1 per 100,000 population to 1,043.0 per 100,000 population (*P* trend <  .001) (Table S2 and Figure 2). The discharge rate of patients aged 36–60 years in 2015 was more than double that in 2005. The age-adjusted discharge rate for pneumonia (12.2 per 100,000 population per year, 95% CI: 9.8–14.7 per 100,000 population per year, *P* trend <  .001) and asthma (1.8 per 100,000 population per year, 95% CI: 1.5–2.1 per 100,000 population per year, *P* trend <  .001) also showed a significant upward trend, while the age-adjusted discharge rate for lung cancer increased moderately (1.3 per 100,000 population per year, 95% CI: 0.03–2.6 per 100,000 population per year, *P* trend =  .046). Furthermore, for patients diagnosed with lung cancer, no clear trends of discharge rate in patients aged both 18–35 years (*P* trend =  .667) and >60 years (*P* trend =  .934) were observed, which differed somewhat from that in patients 36–60 years old (*P* trend <  .001) (Tables S3–S5 and Figure 2). For the four diseases, hospitalization number and mean charge per stay for patients in three severity groups of comorbidities all followed a significant uptrend (*P* trend <  .001) (Tables S6–S9).

**Figure 3:**
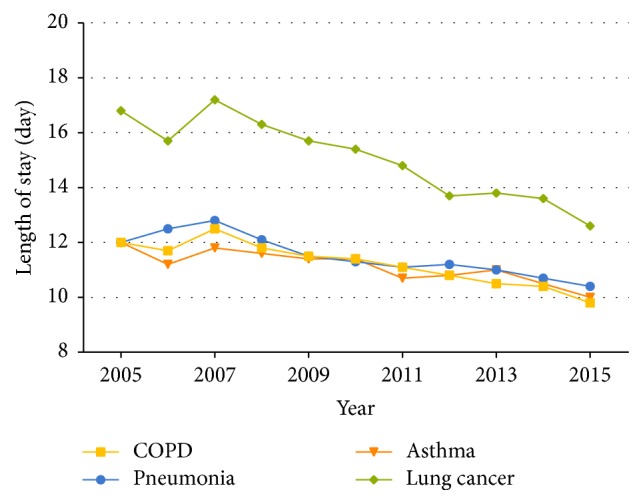
Mean length of stay for hospitalized patients aged ≥18 years with COPD, pneumonia, asthma, and lung cancer, in Dalian city, from 2005 to 2015.

For COPD, the mean LOS showed a significant decline in all age groups and all severity of comorbidities by year (*P* trend <  .001), falling from 12.0 days in 2005 to 9.8 days in 2015. The mean LOS was also influenced by age, with the highest LOS in the age group of >60 years (Table S2 and Figure 3). The age-adjusted mean LOS for patients with pneumonia (0.21 day per year, *P* trend <  .001), lung cancer (0.42 day per year, *P* trend <  .001), and asthma (0.16 day per year, *P* trend <  .001) also showed a significant decrease over time. Asthma and pneumonia showed the same LOS incremental trend by age as COPD (Tables S3–S5 and Figure 3). For pneumonia and lung cancer, the mean LOS followed a significant decline in all severity of comorbidities by year (*P* trend <  .001). Except patients with asthma who had serious comorbidities, the mean LOS in other severity of comorbidities also decreased significantly (*P* trend <  .001) (Tables S6–S9).

**Figure 4:**
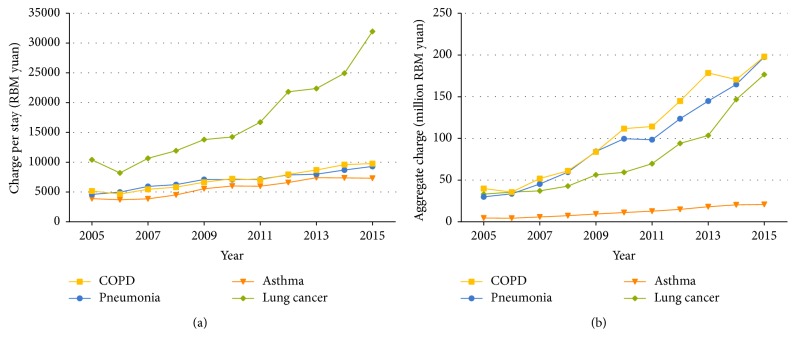
Hospitalization charge for patients aged ≥18 years with COPD, pneumonia, asthma, and lung cancer, in Dalian city, from 2005 to 2015. (a) Mean charge per stay. (b) Aggregate charge.

Mean charge per stay for patients with COPD showed a significant upward trend (*P* trend <  .001), from 5,168 yuan in 2005 to 9,772 yuan in 2015 (Table S2 and Figure 4). Similarly, the mean charge and aggregate charges per stay for COPD patients in all three age groups showed an obvious rising trend (*P* trend <  .001). Mean charge per stay for pneumonia (434.6 yuan per year, *P* trend <  .001), lung cancer (2,113.0 yuan per year, *P* trend <  .001), and asthma (428.0 yuan per year, *P* trend <  .001) also increased significantly in all three age groups (Tables S3–S5 and Figure 4(a)). As a result of the increase in patient number and mean charge per stay, the aggregate charges for COPD, pneumonia, and asthma increased linearly (*R*^2^ = 0.97, 0.97, and 0.98, resp.) and, for lung cancer, exponentially (*R*^2^ = 0.97) (Figure 4(b)). For the four diseases, mean charge per stay for patients under three severity groups of comorbidities all increased significantly (*P* trend <  .001) (Tables S6–S9).

**Figure 5:**
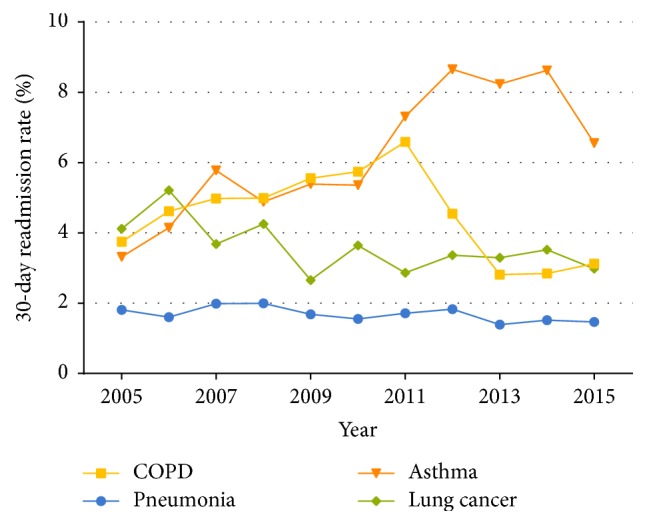
Readmission rate for hospitalized patients aged ≥18 years with COPD, pneumonia, asthma, and lung cancer, in Dalian city, from 2005 to 2015.

There was no clear linear trend for the readmission rate of patients with COPD as the principal diagnosis within 30 days (*P* trend =  .224) and for those with age-specific readmission rates (18–35 years: *P* trend =  .741; 36–60 years: *P* trend =  .437; >60 years: *P* trend =  .090) (Table S2 and Figure 5). The readmission rate of asthma showed a significant increment of 0.46% per year (*P* trend =  .001). By contrast, readmission rates for lung cancer and pneumonia showed a moderate decline (*P* trend  =  .038 and  .044, resp.). Unlike COPD, readmission rates of both pneumonia and asthma patients aged 18–35 years and lung cancer patients aged >60 years declined significantly, whereas that of asthma patients aged >35 years increased (Tables S3–S5 and Figure 5). For asthma, patients without comorbidity showed a significant uptrend in readmission rate. In contrast to asthma, the readmission rate of pneumonia with mild comorbidities and lung cancer without comorbidity decreased moderately (Tables S6–S9).

## 4. Discussion

COPD, pneumonia, lung cancer, and asthma are four prominent respiratory diseases that present medical concerns at treatment level. In this study, we collected the electronic medical records of patients aged ≥18 years from 2005 to 2015 in Dalian, the second largest city in Northeast China. We computed the clinical tracking metrics, hospital discharge rate, readmission rate within 30 days, mean charge per stay, and mean LOS, based on statistical information, to explore the temporal trends of these diseases.

A temporal upward trend of age-adjusted discharge rates was found in all of the studied respiratory diseases, with COPD showing the most rapid improvement (13.45 per 100,000 population per year) in discharge rate for patients ≥18 years from 2005 to 2015. This result differed markedly from those derived from studies conducted in the United States, where the age-adjusted hospital discharge rate of COPD declined significantly (5.29 per 100,000 population per year) for adults aged ≥25 years from 1999 to 2011 [16], while no clear temporal trend was found for patients with COPD or bronchiectasis in patients ≥18 years from 2001 to 2012 (0.20 per 100,000 population per year) [15]. Although great efforts have been made in the past decade by government and communities to forbid smoking in public areas and improve people's awareness of the dangers of smoking, the discharge rate still showed a significant upward trend. The fact that lack of illness prevention awareness and the high-pressure contemporary lifestyle in adults could also contribute to the upward trend should raise the attention of both individuals and the relevant government departments. Regarding age, patients older than 60 years had the highest discharge rate compared with those of other ages, especially for COPD and lung cancer. With the growth of the already large elderly population in China, these four respiratory diseases promise to become a huge burden on society. From another perspective, the health policy “national basic medical insurance” has benefited many more people in China [31], which is also a potential reason for the increment.

Since patients readmitted after discharge usually stay for a longer time in hospitals, readmission rates for patients with diseases are considered to be an important indicator of healthcare quality. In this study, we observed that patients hospitalized with COPD as the primary diagnosis had an average readmission rate of 4.5% for patients aged ≥18 years, whereby there was no significant change in COPD readmission rates between 2005 and 2015. The readmission rate for COPD patients in China is much lower than that of their American counterparts (5.6% versus 7.2%) in the same period from 2009 to 2012 [15], and this can also be seen in elderly patients (4.7% for Chinese patients aged ≥61 years versus 6.8% for American patients aged ≥65 years) from 2008 to 2012 [15, 32]. In contrast to COPD, there has been a significant increment in asthma readmission rates between 2005 and 2015 with an average readmission rate of 6.2%, which is higher than that in the United States [33], especially between 2009 and 2013 (7.0% versus 5.1%). Pneumonia was a kind of acute disease, thus having the lowest readmission rate. With the generalization of family therapy and the development of chronic illness management, the readmission rate of COPD did not show significant trend. For lung cancer, patients who used chemotherapy usually had regular treatment period, and other patients who used target-specific drugs could maintain outpatient treatment. Therefore, patients with lung cancer had lower readmission rate than that of asthma. Different from the pathogenesis of other three diseases, asthma had less differentiating discharge rate in different age groups. Exposure to allergens may provoke asthma, which lead to a higher readmission rate than that of the other three diseases. Severe asthma usually occurred in older people. Condition aggravation and high frequency were important factors of severe asthma. Therefore, the readmission rate of patients with asthma aged >60 years increased significantly. As the readmission rates of asthma have been increasing rapidly and those of COPD have remained at a high level over the past decade, more efforts must be made to optimize postdischarge medication and enhance the community education and intervention for COPD and asthma patients, especially for those in whom COPD and asthma overlap [34].

LOS is one of the major judgment factors for treatment efficiency and level of treatment. The significant reduction in LOS for all four respiratory diseases can be considered as the result of improvements in inpatient treatment in China. Although the magnitude of LOS' decline from 2005 to 2015 was found to be largest in lung cancer in comparison with COPD, pneumonia, and asthma (25.0% versus 18.3%, 13.3%, and 16.7%, resp.), patients with lung cancer still had to stay longer in hospital in 2015 (12.6 days) than patients with COPD, pneumonia, or asthma 10 years ago (12.0 days in 2005), indicating a substantial disease burden. The longest LOS of lung cancer compared with other three diseases may be due to the lengthy chemotherapy period of a subset of patients. For patients with COPD, pneumonia, or asthma, the older the patient is, the longer their stay in hospital is, thus showing a consistent changing pattern by age. Compared with LOS of patients with COPD in the United States, which ranged from 4.5 days in 2001 to 4.0 days in 2012 [15], patients in China with COPD had much longer LOS, although with a higher rate of decrease (0.22 days per year). The difference in hospitalization days may be caused by factors such as the level of health and medication and type of medical insurance. Despite the clear decline and high rate of decline in LOS for COPD patients in China, it is undeniable that a wide gap exists with the United States regarding clinical treatment of COPD. Even within China there exists a difference between different regions. For example, a survey conducted in Guangzhou, the largest city in South China, revealed that the average LOS for 12,838 COPD patients discharged between 2008 and 2014 was 12.04 days [35], which is almost 1 day longer than that for COPD patients included in the present study conducted in Dalian (Northeast China) over the same period.

Despite the significant decrease in LOS, the charge per stay still showed a clear upward trend for all four respiratory diseases, affirming the placement of an increasingly heavy economic burden on the government as well as patients and their families. The charge per stay for hospitalized patients with COPD, pneumonia, and asthma showed a similar changing pattern over the past decade (*P* = .108), and the increment trends were moderate when taking the factor of price rise into consideration. However, lung cancer should be paid more attention, as the charge per stay for hospitalized patients followed a more rapid increment which was almost 5-fold more than that of other three respiratory diseases. This may due to the development of examination techniques and the rising of examination items for lung cancer. For example, PET-CT, enhanced CT examination, and genetic testing methods were used more and more extensively. Furthermore, new chemotherapy drugs (such as pemetrexed) and target-specific drugs for lung cancer appeared in recent years, which also was one reason for the charge increase. In comparison with lung cancer, treatments and examinations of asthma had fewer changes in the past years. For the other three diseases except lung cancer, LOS and charge per stay both increased with the deepening of comorbidities degrees. We also noticed that hospitalization charges were much higher for patients older than 60 years than those ≤60 years for each of the four respiratory diseases, bringing to bear a pressing demand to lower both the LOS and charge per stay for patients >60 years old in order to reduce the total hospitalization burden of the entire population. In addition, hospitalization charges are quite different among different areas in China because of the varying level of economic development. It was reported that the average charge per stay for 723 COPD patients in 6 large cities across China in 2006 was 8,755 yuan [36], which is much higher than that of the 7,021 COPD patients included in the present study (4,766 yuan).

In 2015, smoking prevalence (≥15 years old) was 27.7% in adults, 52.1% among males, and 2.7% among females [37]. Compared with data in 2015, the standardized smoking prevalence in 2010 was 27.4% in adults (≥15 years old), 51.6% among males, and 2.5% among females, indicated by the GATS (Global Adult Tobacco Survey) China survey. The smoking prevalence had a slight increase from 2010 to 2015, and the smoking population increased 15 million during the five years [38]. COPD and lung cancer are two respiratory diseases that have been approved to be influenced by smoke. Similar with the trend of smoking prevalence, the discharge rate of COPD and lung cancer increased significantly from 2010 to 2015. Smoking prevalence among males was much higher than that among females, which was consistent with the number of hospitalizations distribution (Table S1 and Figure 1). Since only two years' official smoking prevalence data were available in China during 2005 to 2015, there was a limitation for a further discussion on temporal trend of clinical tracking metrics influenced by smoke.

There are some limitations to this study in identifying the temporal trend of clinically meaningful metrics. These include the lack of medication and treatment histories and missing details on diseases such as their severity and duration. As a result, we could not include the influence of these factors on LOS, readmission rate, and other metrics. Moreover, the data are mostly representative of the population of one city; therefore, our results and conclusions may not necessarily represent or be generalized to other individuals at a national level.

## 5. Conclusion

In conclusion, from our study on the temporal trends of clinically meaningful statistical information for adult patients with one of four respiratory diseases, we found that the age-adjusted discharge rate increased significantly for any of the four studied diseases. Even though LOS decreased steadily by year, the mean charge per stay and the aggregate charge still increased, even exponentially for lung cancer. These findings are important for administrators, clinicians, and researchers involved in the management of respiratory diseases. They also provide an impression of the severity and importance of related diseases as China gradually enters the “aging society.” Respiratory disease research requires future studies to obtain specific and detailed data from more regions and discover the possible risk factors affecting these hospitalization measures.

## Supplementary Materials

Supplementary Materials Table S1: number of hospitalizations for patients with COPD, pneumonia, asthma, and lung cancer, in Dalian city, from 2005 to 2015. Table S2: age-adjusted and age-specific discharge rate, mean length of stay, mean charge per stay, and readmission rate for patients with COPD aged ≥18 year, in Dalian city, from 2005 to 2015. Table S3: age-adjusted and age-specific discharge rate, mean length of stay, mean charge per stay, and readmission rate for patients with pneumonia aged ≥18 years, in Dalian city, from 2005 to 2015. Table S4: age-adjusted and age-specific discharge rate, mean length of stay, mean charge per stay, and readmission rate for patients with asthma aged ≥18 years, in Dalian city, from 2005 to 2015. Table S5: age-adjusted and age-specific discharge rate, mean length of stay, mean charge per stay, and readmission rate for patients with lung cancer aged ≥18 years, in Dalian city, from 2005 to 2015. Table S6: number of hospitalizations, mean length of stay, mean charge per stay, and readmission rate for patients with COPD aged ≥18 years by the severity of comorbidity, in Dalian city, from 2005 to 2015. Table S7: number of hospitalizations, mean length of stay, mean charge per stay, and readmission rate for patients with pneumonia aged ≥18 years by the severity of comorbidity, in Dalian city, from 2005 to 2015. Table S8: number of hospitalizations, mean length of stay, mean charge per stay, and readmission rate for patients with asthma aged ≥18 years by the severity of comorbidity, in Dalian city, from 2005 to 2015. Table S9: number of hospitalizations, mean length of stay, mean charge per stay, and readmission rate for patients with lung cancer aged ≥18 years by the severity of comorbidity, in Dalian city, from 2005 to 2015.
