# Supplementary material for: Hospitalization Trends in Adult Patients with COPD and Other Respiratory Diseases in Northeast China from 2005 to 2015
Source: Biomed Res Int. 2018 Feb 8;2018:1060497. doi: 10.1155/2018/1060497 (PMC5822913; doi:10.1155/2018/1060497)
Supplement: Supplementary Materials — Table S1: number of hospitalizations for patients with COPD, pneumonia, asthma, and lung cancer, in Dalian city, from 2005 to 2015. Table S2: age-adjusted and age-specific discharge rate, mean length of stay, mean charge per stay, and readmission rate for patients with COPD aged ≥18 year, in Dalian city, from 2005 to 2015. Table S3: age-adjusted and age-specific discharge rate, mean length of stay, mean charge per stay, and readmission rate for patients with pneumonia aged ≥18 years, in Dalian city, from 2005 to 2015. Table S4: age-adjusted and age-specific discharge rate, mean length of stay, mean charge per stay, and readmission rate for patients with asthma aged ≥18 years, in Dalian city, from 2005 to 2015. Table S5: age-adjusted and age-specific discharge rate, mean length of stay, mean charge per stay, and readmission rate for patients with lung cancer aged ≥18 years, in Dalian city, from 2005 to 2015. Table S6: number of hospitalizations, mean length of stay, mean charge per stay, and readmission rate for patients with COPD aged ≥18 years by the severity of comorbidity, in Dalian city, from 2005 to 2015. Table S7: number of hospitalizations, mean length of stay, mean charge per stay, and readmission rate for patients with pneumonia aged ≥18 years by the severity of comorbidity, in Dalian city, from 2005 to 2015. Table S8: number of hospitalizations, mean length of stay, mean charge per stay, and readmission rate for patients with asthma aged ≥18 years by the severity of comorbidity, in Dalian city, from 2005 to 2015. Table S9: number of hospitalizations, mean length of stay, mean charge per stay, and readmission rate for patients with lung cancer aged ≥18 years by the severity of comorbidity, in Dalian city, from 2005 to 2015. [file 1060497.f1.pdf]

Table S1. Number of hospitalizations for patients with COPD, pneumonia, asthma and lung cancer, in Dalian city, from 2005 to 2015.

	2005	2006	2007	2008	2009	2010	2011	2012	2013	2014	2015
All	351754	373436	454327	490004	554416	617817	677951	756058	817872	919441	955713
Gender											
Male	177227	186110	223246	241403	271405	302349	329720	362408	390912	NA	NA
Female	174527	187324	231081	248599	283010	315468	348231	393647	416960	NA	NA
Age, ≥18 y	321478	339916	417778	451719	515296	571872	631078	706074	757044	846431	885771
Gender											
Male ≥18y	158868	165832	201360	218024	247623	274703	301443	332372	360100	NA	NA
Female ≥18y	162610	174084	216418	233695	267673	297169	329635	373702	396944	NA	NA
COPD											
Age ≥18y	7642	7021	9359	10062	11663	14509	15006	18106	19224	18653	20965
Male ≥18y	4483	4079	5251	5521	6404	7997	8198	9961	10675	NA	NA
Female ≥18y	3159	2942	4108	4541	5259	6512	6808	8145	8549	NA	NA
Pneumonia											
Age ≥18y	5767	5374	6297	7766	9555	10948	10976	12200	14435	14963	15757
Male ≥18y	3352	3096	3796	4429	5438	6057	6088	6591	7651	NA	NA
Female ≥18y	2415	2278	2501	3337	4117	4891	4888	5609	6784	NA	NA
Asthma											
Age ≥18y	1115	1158	1471	1597	1707	1829	2065	2207	2283	2620	2640
Male ≥18y	498	470	605	677	720	735	859	912	894	NA	NA
Female ≥18y	617	688	866	919	987	1094	1206	1295	1389	NA	NA
Lung Cancer											
Age ≥18y	3500	4277	3936	4020	4326	4473	4679	5024	5462	6503	6709
Male ≥18y	2336	2773	2569	2537	2719	2820	2947	3021	3282	NA	NA
Female ≥18y	1164	1504	1367	1483	1607	1653	1732	2003	2180	NA	NA

Table S2. Age-adjusted and age-specified discharge rate, mean length of stay, mean charge per stay and readmission rate for patients with COPD aged ≥18 years, in Dalian city, from 2005 to 2015.

Year	2005	2006	2007	2008	2009	2010	2011	2012	2013	2014	2015	P Trend
Rate per 100,000												
All Discharges #	149.0	133.0	172.5	179.3	203.9	226.9	226.0	258.3	263.1	245.4	264.7	<0.001
Age Group, year												
18–35	8.3	7.3	9.0	9.5	11.2	12.1	13.0	15.0	15.1	14.0	14.4	<0.001
36–60	55.4	48.8	65.6	73.9	95.6	108.9	122.8	132.3	137.4	133.4	137.4	<0.001
>61	647.1	579.6	746.1	759.5	831.6	919.0	873.8	1020.7	1033.2	949.1	1043.0	<0.001
Length of Stay, day												
All Discharges #	12.0	11.7	12.5	11.8	11.5	11.4	11.1	10.8	10.5	10.4	9.8	<0.001
Age Group, year												
18–35	11.4	10.1	12.2	10.6	10.2	10.2	9.3	9.1	9.5	9.6	8.6	0.003
36–60	12.2	12.4	12.7	12.3	12.2	12.1	12.0	11.6	10.9	10.7	10.3	<0.001
>61	12.6	12.7	12.6	12.3	12.1	11.8	11.6	11.4	11.2	11.1	10.7	<0.001
Charge per Stay (RMB Yuan)												
All Discharges #	5168	4677	5466	5821	6647	7228	7037	7953	8707	9594	9772	<0.001
Age Group, year												
18–35	4886	4566	5646	6647	6935	7644	7480	8799	9789	11660	12178	<0.001
36–60	5215	4478	5170	4994	6062	6592	6325	7171	7469	8144	8253	<0.001
>61	5523	5414	5969	6681	7763	8266	8235	8655	10261	10043	9830	<0.001
Readmission rate (%)												
All Discharges $	3.75	4.61	4.98	4.99	5.56	5.74	6.59	4.55	2.81	2.85	3.13	0.224
Age Group, year												
18–35	0.75	1.69	3.55	0.00	0.56	2.99	1.37	2.81	1.20	0.00	1.34	0.741
36–60	3.70	4.35	7.59	7.59	9.42	11.11	11.28	7.07	1.97	2.69	2.23	0.437
>61	4.01	5.02	4.74	4.78	5.00	4.77	5.86	4.16	3.18	3.04	3.50	0.090

Age-adjusted estimates; $ Unadjusted for age

Table S3. Age-adjusted and age-specified discharge rate, length of stay, mean charge per stay and readmission rate for patients with pneumonia aged ≥18 years, in Dalian city, from 2005 to 2015.

Year	2005	2006	2007	2008	2009	2010	2011	2012	2013	2014	2015	P Trend
Rate per 100,000												
All Discharges	112.3	102.2	116.8	140.5	170.5	175.8	171.0	182.1	213.6	215.9	215.6	<0.001
Age Group, y												
18-35	49.4	38.5	31.7	58.8	78.2	74.0	68.0	75.6	130.7	119.7	79.5	0.004
36-60	58.1	55.8	58.9	78.3	106.3	102.6	112.0	114.4	137.0	151.0	147.7	<0.001
≥61	368.8	338.8	421.3	451.3	505.1	551.4	509.3	550.6	566.4	559.3	634.7	<0.001
Length of Stay, d												
All Discharges #	12.0	12.5	12.8	12.1	11.5	11.3	11.1	11.2	11.0	10.7	10.4	<0.001
Age Group, y												
18-35	11.0	11.8	12.0	11.2	10.4	10.8	10.4	10.5	10.5	10.0	9.8	0.001
36-60	12.5	12.6	13.0	12.3	11.6	11.3	11.0	11.2	11.0	10.7	10.4	<0.001
≥61	12.6	13.5	13.7	13.3	13.3	12.4	12.5	12.3	11.9	11.8	11.4	0.001
Charge per Stay (RMB yuan)												
All Discharges #	4596	4987	5948	6253	7104	7100	7162	7880	8003	8695	9296	<0.001
Age Group, y												
18-35	3601	3651	4173	4576	5236	5596	5490	5873	6115	6215	6761	<0.001
36-60	4588	4772	6031	6025	6743	6518	6781	7392	7365	8189	8737	<0.001
≥61	6320	7865	8759	9747	11293	11274	11072	12652	12986	14326	15167	<0.001
Readmission rate (%)												
All Discharges $	1.81	1.60	1.99	2.00	1.68	1.55	1.71	1.83	1.39	1.52	1.47	0.044
Age Group, y												
18-35	0.51	0.00	0.20	0.00	0.32	0.72	0.09	0.23	0.27	0.21	0.64	0.46
36-60	1.01	0.83	1.75	1.78	0.56	0.63	0.54	0.59	0.44	0.67	0.42	0.04
≥61	2.52	2.30	2.35	2.58	2.68	2.18	2.67	2.74	2.22	2.32	2.06	0.36

Age-adjusted estimates; $ Unadjusted for age

Table S4. Age-adjusted and age-specified discharge rate, length of stay, mean charge per stay and readmission rate for patients with asthma aged ≥18 years, in Dalian city, from 2005 to 2015.

Year	2005	2006	2007	2008	2009	2010	2011	2012	2013	2014	2015	P Trend
Rate per 100,000												
All Discharges [#]	21.8	22.2	27.6	29.2	30.8	30.1	33.3	34.8	35.2	39.6	39.7	<0.001
Age Group, y												
18-35	6.5	6.6	7.1	8.1	7.3	5.6	7.7	8.3	8.0	9.8	11.0	<0.001
36-60	24.1	24.8	30.5	31.7	35.1	36.7	39.6	42.9	43.0	47.5	47.8	<0.001
≥61	41.4	41.7	54.4	58.4	59.3	53.9	59.5	57.8	60.2	68.7	66.2	<0.001
Length of Stay, d												
All Discharges [#]	12.0	11.2	11.8	11.6	11.4	11.4	10.7	10.8	11.0	10.5	10.0	<0.001
Age Group, y												
18-35	9.4	8.6	10.2	10.2	9.2	9.2	8.4	8.6	9.5	8.8	8.5	0.15
36-60	13.1	11.9	11.9	11.8	12.0	12.1	11.2	11.3	11.3	10.8	10.4	<0.001
≥61	13.5	13.5	14.0	13.4	13.3	13.2	13.1	13.3	13.0	12.5	11.2	0.004
Charge per Stay (RMB yuan)												
All Discharges [#]	38945	3704	3862	4509	5561	6020	5982	6595	7412	7368	7332	<0.001
Age Group, y												
18-35	2746	3140	3188	3734	5025	5664	5324	5809	6215	6464	5914	<0.001
36-60	4159	3800	3914	4588	5738	5912	6054	6627	7157	7278	7759	<0.001
≥61	5133	4404	4871	5619	5994	6928	6907	7853	10161	9162	8581	<0.001
Readmission rate (%)												
All Discharges [$]	3.32	4.15	5.78	4.88	5.40	5.36	7.31	8.65	8.23	8.63	6.55	0.001
Age Group, y												
18-35	5.00	2.88	5.50	3.97	2.59	1.06	0.76	1.43	0.00	1.27	0.58	0.001
36-60	3.15	3.03	3.84	3.37	3.69	4.43	5.67	5.71	6.66	6.40	5.47	<0.001
≥61	3.47	6.86	9.88	7.99	9.52	8.51	12.95	17.96	14.23	15.31	10.30	0.007

Age-adjusted estimates; $ Unadjusted for age

Table S5. Age-adjusted and age-specified discharge rate, length of stay, mean charge per stay and readmission rate for patients with lung cancer aged ≥18 years, in Dalian city, from 2005 to 2015.

Year	2005	2006	2007	2008	2009	2010	2011	2012	2013	2014	2015	P Trend
Rate per 100,000												
All Discharges #	68.3	81.3	72.8	72.1	76.2	70.6	71.2	73.2	77.0	88.5	89.5	0.046
Age Group, y												
18-35	1.7	2.5	2.0	2.4	2.5	1.7	1.5	1.6	2.0	3.0	2.4	0.667
36-60	39.6	52.7	44.5	46.3	53.4	47.7	50.5	54.2	58.8	68.0	74.3	<0.001
≥61	261.2	294.8	271.7	262.1	265.1	251.6	247.5	248.2	255.3	290.9	280.2	0.934
Length of Stay, d												
All Discharges #	16.8	15.7	17.2	16.3	15.7	15.4	14.8	13.7	13.8	13.6	12.6	<0.001
Age Group, y												
18-35	17.9	14.0	16.3	16.3	13.0	15.3	12.5	12.2	13.7	12.9	10.4	0.003
36-60	16.2	16.5	18.0	16.3	17.1	15.4	16.2	14.4	13.8	14.0	13.6	0.001
≥61	16.3	16.5	16.8	16.1	16.7	15.8	15.1	14.4	13.8	13.8	13.5	<0.001
Charge per Stay (RMB yuan)												
All Discharges #	10421	8208	10666	11935	13808	14251	16730	21832	22370	24944	31958	<0.001
Age Group, y												
18-35	10290	6560	9912	11387	12097	13207	16769	24553	23116	23125	36544	<0.001
36-60	10803	9090	11522	12700	15153	15211	17486	21275	23561	27389	31826	<0.001
≥61	9597	8604	9603	10771	13038	13401	14586	18709	17819	21338	24476	<0.001
Readmission rate (%)												
All Discharges $	4.11	5.21	3.68	4.25	2.66	3.64	2.86	3.36	3.30	3.524	2.98	0.038
Age Group, y												
18-35	0.00	2.50	3.13	0.00	0.00	0.00	0.00	0.00	3.13	2.08	0.00	0.916
36-60	2.41	3.78	2.87	2.54	2.18	2.47	1.56	2.54	2.21	3.11	2.25	0.337
≥61	5.15	6.39	4.25	5.41	3.06	4.44	3.65	3.96	3.99	3.92	3.51	0.026

Age-adjusted estimates; $ Unadjusted for age

Table S6. Number of hospitalizations, mean length of stay, mean charge per stay and readmission rate for patients with COPD aged ≥18 years by the severity of comorbidity, in Dalian city, from 2005 to 2015.

Year	2005	2006	2007	2008	2009	2010	2011	2012	2013	2014	2015	P Trend
Number of hospitalizations												
Severity of comorbidity												
without comorbidity	5144	4400	6049	6476	7355	8897	9154	10694	11240	9722	11111	<0.001
mild comorbidities	2008	2044	2659	2887	3375	4553	4705	5995	6499	6628	7797	<0.001
serious comorbidities	490	577	651	699	933	1059	1147	1417	1485	2303	2057	<0.001
Length of Stay, d												
Severity of comorbidity												
without comorbidity	12.0	12.3	12.2	11.9	11.8	11.8	11.5	11.3	10.9	10.8	10.3	<0.001
mild comorbidities	13.4	12.9	12.9	12.7	12.5	11.8	11.8	11.4	11.3	11.1	10.7	<0.001
serious comorbidities	14.1	13.8	14.9	13.9	13.0	13.0	12.3	12.5	12.4	11.9	11.3	<0.001
Charge per Stay (RMB Yuan)												
Severity of comorbidity												
without comorbidity	4875	4893	5256	5741	6566	7243	7153	7779	8228	8713	9086	<0.001
mild comorbidities	6482	5697	6397	7137	8506	8444	8462	8636	8742	9994	9590	<0.001
serious comorbidities	7033	5696	8181	8148	9145	10396	9340	10744	10783	12328	11252	<0.001
Readmission rate (%)												
Severity of comorbidity												
without comorbidity	3.88	4.41	5.71	5.51	6.41	7.36	8.02	5.07	2.52	2.70	2.59	0.291
mild comorbidities	3.99	5.96	4.07	4.56	4.30	4.07	5.26	4.44	3.24	2.90	3.37	0.059
serious comorbidities	3.59	4.15	5.68	5.75	7.61	4.44	6.90	3.81	4.21	3.97	6.25	0.819

Table S7. Number of hospitalizations, mean length of stay, mean charge per stay and readmission rate for patients with pneumonia aged ≥18 years by the severity of comorbidity, in Dalian city, from 2005 to 2015.

Year	2005	2006	2007	2008	2009	2010	2011	2012	2013	2014	2015	P Trend
Number of hospitalizations												
Severity of comorbidity												
without comorbidity	3540	3142	3470	4524	5619	6151	6150	6676	8250	8158	7961	<0.001
mild comorbidities	1492	1528	1862	2185	2751	3276	3215	3766	4243	4642	5352	<0.001
serious comorbidities	735	704	965	1057	1185	1521	1611	1758	1942	2163	2444	<0.001
Length of Stay, d												
Severity of comorbidity												
without comorbidity	11.9	12.5	12.7	11.9	11.7	11.3	11.2	11.2	11.1	10.8	10.6	<0.001
mild comorbidities	12.9	13.3	13.8	13.9	13.1	12.5	12.5	12.4	11.9	11.6	11.2	<0.001
serious comorbidities	13.5	14.6	14.7	14.0	14.2	13.2	13.0	12.4	12.0	12.1	11.6	<0.001
Charge per Stay (RMB Yuan)												
Severity of comorbidity												
without comorbidity	4467	5294	6276	6198	7366	7232	7403	8724	8515	9592	11131	<0.001
mild comorbidities	6562	7091	8429	9916	10850	10646	10856	12174	11979	12592	13442	<0.001
serious comorbidities	7884	10329	10941	12035	12741	14183	12393	12888	13974	15191	15836	<0.001
Readmission rate (%)												
Severity of comorbidity												
without comorbidity	1.37	1.00	1.61	1.69	1.04	1.00	0.99	1.40	0.98	0.85	0.89	0.073
mild comorbidities	2.40	2.62	2.48	2.15	2.96	2.31	2.29	2.09	1.46	2.04	1.75	0.018
serious comorbidities	2.94	2.33	2.66	3.32	2.07	2.36	3.60	3.17	3.19	3.10	2.91	0.266

Table S8. Number of hospitalizations, mean length of stay, mean charge per stay and readmission rate for patients with asthma aged ≥18 years by the severity of comorbidity, in Dalian city, from 2005 to 2015.

Year	2005	2006	2007	2008	2009	2010	2011	2012	2013	2014	2015	P Trend
Number of hospitalizations												
Severity of comorbidity												
without comorbidity	924	942	1173	1264	1250	1395	1569	1641	1674	1834	1842	<0.001
mild comorbidities	171	183	244	290	391	375	412	464	518	662	648	<0.001
serious comorbidities	20	33	54	43	66	59	84	102	91	124	150	<0.001
Length of Stay, d												
Severity of comorbidity												
without comorbidity	12.7	11.8	12.3	12.2	12.1	12.4	11.6	11.8	11.9	11.3	10.6	0.004
mild comorbidities	14.0	14.4	13.3	12.4	12.7	11.9	11.6	11.4	11.6	11.2	10.4	<0.001
serious comorbidities	13.0	12.4	14.5	12.9	14.1	13.9	13.9	13.5	11.3	12.0	10.8	0.091
Charge per Stay (RMB Yuan)												
Severity of comorbidity												
without comorbidity	4064	3659	3987	4710	5409	5950	5919	6260	7974	7325	7565	<0.001
mild comorbidities	5436	4884	4578	5336	6428	6785	7300	8673	8667	8694	8608	<0.001
serious comorbidities	7240	5357	6299	6287	7691	7738	8087	10081	9186	13076	9483	0.002
Readmission rate (%)												
Severity of comorbidity												
without comorbidity	3.01	4.43	6.25	5.25	6.11	6.73	8.88	11.10	10.79	11.29	7.85	<0.001
mild comorbidities	5.56	3.98	6.55	5.07	4.27	2.74	3.78	4.98	5.07	5.58	4.52	0.739
serious comorbidities	5.26	3.13	1.89	2.38	6.45	0.00	10.53	5.15	0.00	4.20	7.91	0.558

Table S9. Number of hospitalizations, mean length of stay, mean charge per stay and readmission rate for patients with lung cancer aged ≥18 years by the severity of comorbidity, in Dalian city, from 2005 to 2015.

Year	2005	2006	2007	2008	2009	2010	2011	2012	2013	2014	2015	P Trend
Number of hospitalizations												
Severity of comorbidity												
without comorbidity	1948	2334	1940	2020	2169	2273	2511	2463	2591	2878	3154	<0.001
mild comorbidities	432	470	493	508	590	605	657	659	725	993	1026	<0.001
serious comorbidities	1120	1473	1503	1492	1567	1595	1511	1902	2146	2632	2529	<0.001
Length of Stay, d												
Severity of comorbidity												
without comorbidity	16.2	16.2	17.1	15.7	16.1	15.2	15.6	13.8	13.9	13.9	13.4	<0.001
mild comorbidities	16.5	16.7	17.1	17.3	16.8	16.4	15.5	15.8	14.4	14.1	14.3	<0.001
serious comorbidities	16.5	16.8	17.3	16.5	17.7	16.1	15.0	14.6	13.6	13.7	13.4	<0.001
Charge per Stay (RMB Yuan)												
Severity of comorbidity												
without comorbidity	10421	8897	10545	12196	14716	15058	17642	22963	24175	29193	34871	<0.001
mild comorbidities	10130	8999	10845	12398	14127	14867	15836	23401	22157	27106	27847	<0.001
serious comorbidities	8448	7985	9044	9377	11599	11294	11003	13089	12709	14611	15679	<0.001
Readmission rate (%)												
Severity of comorbidity												
without comorbidity	4.79	8.76	4.08	4.50	2.41	2.80	2.53	2.80	2.41	2.02	1.74	0.007
mild comorbidities	4.35	9.56	4.01	4.96	3.69	6.89	3.14	4.11	3.72	3.65	3.32	0.116
serious comorbidities	3.42	11.00	3.44	4.19	2.82	4.04	3.56	4.16	4.53	5.49	4.68	0.638
